# Going With the Glow: How Bacteria Integrate Molecular Signals to Synchronize Bioluminescence

**DOI:** 10.1371/journal.pbio.1000076

**Published:** 2009-03-24

**Authors:** Mary Hoff

After a long day in the microbiology lab, Vibrio harveyi may just want to relax, but if enough of its neighbors are game for a group project, it just can't say no. V. harveyi is a bioluminescent marine bacterium that uses a chemical peer-pressure process called quorum sensing to determine whether to emit light and carry out other collective activities. Quorum sensing, which occurs in other bacteria as well, is both fascinating in itself and instructive for an array of disciplines from entomology to robotics. It goes like this: Quorum-sensing bacteria release small molecules called autoinducers. These molecules convey the presence of the cells to neighboring bacteria. When enough autoinducer molecules float around in the extracellular medium, the bacteria, sensing a critical mass, produce a synchronized response—in V. harveyi's case, this response is a group glow.

Quorum sensing in bacteria that respond to a single autoinducer is understood fairly well to cause changes in gene expression that are induced by the accumulated autoinducer. V. harveyi, however, uses three autoinducers to get its herd mentality message across. The three autoinducers, called AI-1, CAI-1, and AI-2, are detected by the transmembrane receptors LuxN, CqsS, and LuxPQ, respectively, which then inhibit the transcription of genes whose products, in turn, block the production of another protein, LuxR. When the autoinducer concentration “tipping point” is reached, gene transcription is inhibited sufficiently and thus LuxR is produced, causing the bacteria to glow.

How do the multiple autoinducers work together to stimulate LuxR production? And what's the evolutionary value of toting multiple triggers? Tao Long, Bonnie Bassler, Ned Wingreen, and their Princeton colleagues discovered answers to both of those questions using single-cell fluorescence microscopy to track the response of individual genetically engineered bacteria to various combinations and quantities of autoinducers.

Since previous research had shown that CAI-1 plays a relatively minor role in the overall autoinduction scheme of things, the researchers started off simplifying things a bit by engineering a strain of V. harveyi that lacked the CqsS pathway. As a way to keep track of what was happening in the cells, they also inserted genes for green fluorescent protein (GFP) and red fluorescent protein (mCherry), such that GFP would produce a green glow when LuxR production was blocked, and mCherry would produce a red color whenever transcription of any sort was taking place (providing a normalizer that could be used to control for cell size, cell cycle stage, illumination, etc.). They then further engineered three separate strains derived from this engineered bacterium: LuxN+, which responds only to AI-1; LuxPQ+, which responds only to AI-2; and LuxN+ LuxPQ+, which responds to both AI-1 and AI-2.

Exposure of the three strains to various combinations of autoinducers was illuminating. In the LuxN+ strain, the strength of the LuxR production blockage (as indicated by the strength of the GFP fluorescence normalized against mCherry) decreased with increasing amounts of AI-1, meaning that the pathway inhibiting V. harveyi's fluorescence response was weakening. Similarly, when the LuxPQ+ mutant was exposed to varying levels of AI-2, the lower the dose of autoinducer, the more the bacteria glowed GFP green. In addition, when the LuxN+LuxPQ+ strain was exposed to one or the other, but not both, autoinducers, GFP expression was only partly inhibited.

**Figure:**
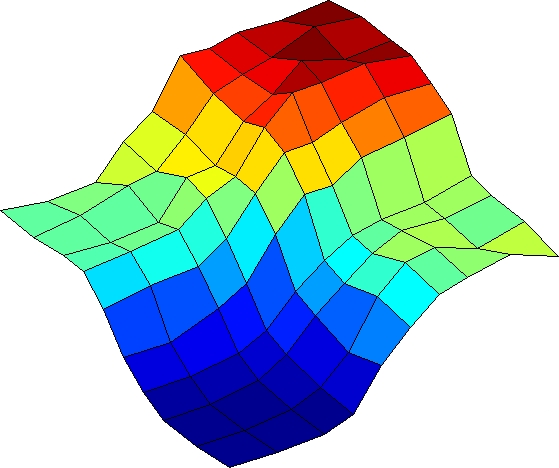
Quorum-sensing response in an engineered strain of V. harveyi, measured by GFP, reveals a strictly additive response to two distinct intercellular signals, AI-1 and AI-2. The symmetry of the response surface shows that the two chemical signals receive nearly equal weights.

The researchers then tested the effects of various combinations of AI-1 and AI-2 levels on individual cells of the LuxN+LuxPQ+ strain. Not surprisingly, lower combined autoinducer levels corresponded to higher GFP expression, and vice versa. In an unexpected outcome, the low AI-1/high AI-2 combination yielded virtually identical results to low AI-2/high AI-1—and the two inputs were strictly additive. The researchers also found that, unlike other bacterial regulatory circuits that show considerable variability among individuals, the induction of fluorescence was strikingly similar among all individuals in a population, although there was slightly more cell-to-cell variation in the LuxPQ+ strain than in the LuxN+ strain.

Why does V. harveyi have multiple autoinducers that essentially act in the same way via the same pathway? One possible explanation is that the multiple autoinducers could reveal information about what other bacterial species might be present and in what relative quantities, since CAI-1 is also released by V. harveyi relatives, and AI-2 is released by a wide range of bacterial species. However, the fact that, in this study, high AI-1/low AI-2 and low AI-1/high AI-2 caused identical responses suggests that this is not the case. Rather, the researchers hypothesize that different combinations of autoinducers might be characteristic of particular stages of community development. If so, multiple signals could make it possible for bacterial populations to induce tightly synchronized quorum-sensing responses, while at the same time allowing them to show unique characteristics at different developmental stages.

